# Effects of Sex on Intra-Individual Variance in Urinary Solutes in Stone-Formers Collected from a Single Clinical Laboratory

**DOI:** 10.1371/journal.pone.0053637

**Published:** 2013-06-19

**Authors:** Guy M. L. Perry, Steven J. Scheinman, John R. Asplin

**Affiliations:** 1 Department of Medicine, SUNY Upstate Medical University, Syracuse, New York, United States of America; 2 Litholink Corporation, Chicago, Illinois United States of America; Fondazione IRCCS Ospedale Maggiore Policlinico & Fondazione D'Amico per la Ricerca sulle Malattie Renali, Italy

## Abstract

**Background/Aims:**

Our work in a rodent model of urinary calcium suggests genetic and gender effects on increased residual variability in urine chemistries. Based on these findings, we hypothesized that sex would similarly be associated with residual variation in human urine solutes. Sex-related effects on residuals might affect the establishment of physiological baselines and error in medical assays.

**Methods:**

We tested the effects of sex on residual variation in urine chemistry by estimating coefficients of variation (*CV*) for urinary solutes in paired sequential 24-h urines (≤72 hour interval) in 6,758 females and 9,024 males aged 16–80 submitted to a clinical laboratory.

**Results:**

Females had higher *CV*s than males for urinary phosphorus overall at the False Discovery Rate (*P*<0.01). There was no effect of sex on *CV* for calcium (*P*>0.3). Males had higher *CV*s for citrate (*P*<0.01) from ages 16–45 and females higher *CV*s for citrate (*P*<0.01) from ages 56–80, suggesting effects of an extant oestral cycle on residual variance.

**Conclusions:**

Our findings indicate the effects of sex on residual variance of the excretion of urinary solutes including phosphorus and citrate; differences in *CV* by sex might reflect dietary lability, differences in the fidelity of reporting or genetic differentiation in renal solute consistency. Such an effect could complicate medical analysis by the addition of random error to phenotypic assays. Renal analysis might require explicit incorporation of heterogeneity among factorial effects, and for sex in particular.

## Introduction

‘Physiological’ traits [1] such as renal solute excretion frequently have high proportions of residual (error) variance: there is high inter- and intra-individual variation in urinary excretion of phosphate, urate, citrate, sodium, oxalate [2], [3], [4] and albumin [5]. Residual variance in oxalate excretion is higher among calcium oxalate stone formers than non-formers [6]; intra-individual variation (IIV) in 24-h calcium excretion is higher in children with nocturnal enuresis, sometimes associated with hypercalciuria, compared to controls [7], [8]. It has been argued from these phenomena that single 24-hour urine collections are insufficient for the evaluation of risk factors for kidney stones; a sample of 1142 individuals collected from a private urology practice and a stone research clinic found that in 68% of cases paired urine measurements for stone disease were sufficiently different by a value large enough to produce misdiagnosis if diagnosis had been based only on a single measurement [9].

There is an accumulating body of evidence that residual variability in phenotype may be under partial genetic control [10], [11], [12], [13]. There are examples of sexual modification of residual variance or genetic effects on residual variance, including *Drosophila* morphology [14], environmental resistance in fish [10] and rodent models of behavior [15]. Variation in serum lipids and apolipoprotein concentrations is higher in women over long (>6 month) intervals [16]. Environmental buffering of phenotype in mice also appears to be sex-specific [17]. Using a rodent model of hypercalciuria, the Genetic Hypercalciuric Stone-forming (GHS) rat, we have detected several sex-specific loci associated with absolute-transformed residuals in urinary calcium [13], a model of hypercalciuria and kidney stone disease. Sex-related differences in human urinary calcium and stone formation are well established [18], [19], and there is also substantial residual variance in renal solute excretion [2], [3], [5], [6], [7], [8], [9]. Clarifying sexual biases in residual variance could improve analysis and diagnosis of urinary solute patterns in renal disease.

Given sex and genetic effects on residual variance, we hypothesized that *CV*s in urinary solute excretion, including urinary calcium, would also differ between men and women in a sample of 15,782 kidney stone-formers (6,758 females and 9,024 males) collected by Litholink Inc. (Chicago, IL), a commercial assay company. *A priori* predictions of direction (higher *CV*s in males or females) were not possible since sex differences in residual variation of urinary calcium in our rat model indicated sex-by-strain interaction [13], so that only an effect of sex was predicted rather than direction. Sex-limited effects on random residuals might affect medical analysis and/or diagnosis; intra-individual variance (IIV) in renal traits may be of great diagnostic significance [9].

## Materials and Methods

### Sample collection

Paired 24-hour urines with a maximum interval of 72 hours were obtained as part of routine clinical testing for 7,372 female and 9,780 male kidney stone patients and submitted to a single reference laboratory (Litholink Corp) for urine chemistry measurement. Urines were collected at room temperature using an antimicrobial preservative. Individual 24-h urines were measured for all subjects for calcium (Ca; mg), citrate (Cit; mg), chloride (Cl; mmoles), creatinine (Cr; mg), potassium (K; mmoles), sodium (Na; mmoles), magnesium (Mg; mg), oxalate (Ox; mg), phosphorus (P; g), ammonium (NH_4_; mmoles), sulfate (SO_4_; meq), uric acid (UA; g) and urea nitrogen (UN; mg). Cr was collected for the purposes of measuring completeness of collection, and Na and UN for the purposes of controlling for dietary inputs of salt and protein. Where a measurement was missing for any urinary solute, that individual was removed from all analysis for that urinary trait. Weight (kg), age (yrs) and sex were collected for each individual but no additional clinical or background information was collected by Litholink Inc. Individuals known to be receiving treatment for stones at the time of collection were excluded from the study, although not all patients had a fully-known treatment history. Use of this data set was approved by the Western Institutional Review Board (Olympia, WA). Records were collected over the history of Litholink's operation. No experimentation was performed.

### Differences in univariate coefficients of variance (*CV*) by sex

Coefficients of variation (*CV*) for paired 24-h measurements of urinary excretion rates were estimated for all individuals as 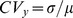. *CV*s for each trait were ranked in ascending order *x*_1_…*x*_n_ for independent analysis Ranked *CV*s were used to avoid complications from non-normal distributions in this nonparametric statistic while still permitting multivariate models to account for the effects of significant covariates. Global effects on *CV* for each urinary solute were estimated simultaneously with covariate selection using multivariate backwards regression in SAS [20] with elimination of nonsignificant variables at *P* <0.1, beginning with the starting model (Model 1):
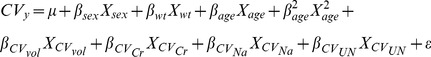
where 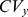 was the coefficient of variation ranking for urinary variable *y*, 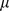 was the model intercept, 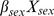 are the coefficient of (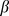) and incident vector for (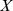) the effect of sex on *CV_y_*, 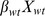 is the effect of weight (kg) on *CV_y_*, 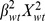 is the effect of higher-order *CV*-weight interactions on *CV_y_*, 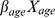 is the linear effect of age on *CV_y_* and 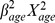 the effect of higher-order curvature age-*CV_y_* relationships to account for changes in CV within or across life history categories, 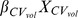 was the effect of ranked *CV* for differences in urinary volume between the two samples, 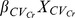 was the effect of ranked 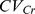 on *CV_y_*, 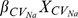 was the effect of ranked 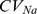 on *CV_y_*, 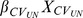 was the effect of ranked 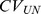 and 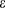 was error. Colinearity among the covariates (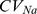, 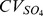 and 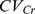) was tested in PROC REG prior to further analysis to prevent biased control of dietary and collection effects on urinary output by effective duplication of closely connected effects. Where variance inflation functions (VIF) exceeded 10.0 for a pair of linear and quadratic terms for the same covariate, the quadratic term was excluded from the model. The final complement of regression terms included via backwards regression normally ranged from three to five (Table S1). Multiple regression for linear covariates was preferred to the establishment of narrow factorial categories in order to reduce degrees of freedom lost on subcategorizations [21]; our single experiment involving age categories was based on explicit life-history contrasts (see below). Sex was coded as a binary variable with female indicated as 0 and males as 1, so that positive, significant values for 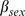 indicated high ranked variance for males and negative values high ranked variance in females. All individuals with 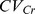 scores above the upper 30^th^ percentile were excluded prior to analysis as possibly irreparably biased due to errors of collection. Ranked 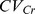 was included to account for collection variance for all traits in the remaining individuals, and 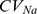 and 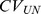 were included as covariates to account for dietary protein and salt on those traits [22]. Mean *CV* for each trait by sex was estimated using a least-squares (LS) model based on terms retained from backward stepwise regression, above, with sex coded as a fixed effect and all other terms as partial regressions [20].

The significance of sex effects on *CV*s were evaluated using the false discovery rate (FDR) [23]. Nominal *P*-values (*P*_nom_) for sex effects on *CV* were ranked in ascending order from 1 to *m* (the total number of traits). Where the ranked *P*_i_ for hypothesis *H*_i_ for each trait *i* satisfied the relation *P*_i_≤*αi*/*m*, where *α* was the nominal *a priori* significance threshold ( = 0.05), we rejected the null hypothesis of no significant difference in *CV* between males and females for that test and all tests with lower *P*_i_ [23].

### Sex differences in urinary *CV* by major age categories in female life-history

In order to test the effects of post-menarche *vs*. post-menopausal female physiology on *CV*s for urinary traits, we divided the population into two classes: 1) a rough ‘menarche-menopause’ (MM) category of 16–45 years and 2) a rough ‘post-menopausal’ (PM) group of 56–80 years. These age categories were selected to coincide with the approximate mean age of inception of menopause in Western women [24] with a ten-year interval window to limit overlap of approximate menstrual- and menopausal-age individuals outside their respective categories. The effects of sex and other factors on urinary *CV*s was then tested using backwards regression (*P*_thr_<0.1) in Model 1 as above, within each of categories 1) and 2). Significance thresholds were estimated separately within each group at the FDR as above.

We next tested for interactive effects of age-group with sex on residual variance again using backwards stepwise regression for joint covariate selection and analysis, starting with the saturated general linear model (Model 2)
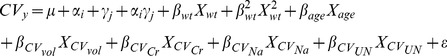
where 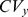 was the coefficient of variation ranking for urinary variable *y*, 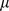 was the model intercept, 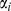 is the effect of sex *i* on the *CV* for trait *y*, 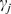 is the effect of age group *j*, 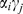 is the effect of interaction between sex *i* and age group *j* on *CV_y_* and all other terms were as per Model 1 (above). As above, from three to five terms were normally included in the final analysis via backwards regression (Table S1). The significance of sex×age class interaction was corrected using the FDR [23]. Covariates were removed as required by VIF conditioning as in Model 1. Least-squares estimates of means and standard errors by sex and age-class were determined in a general linear model in PROC GLM [20] to estimate mean *CV* ranks while avoiding contamination from other dependent variables.

## Results

### Means and *CV*s

After excluding individuals in the upper 30^th^ percentile of 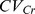, a total of 9,024 males and 6,758 females remained for analysis. *CV*s were highest overall for Na and Cl, and lowest for Cr although the range of individual *CV*s was highest for Ca (Table 1).

**Table 1:** Mean coefficients of variation (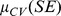) and minimum and maximum *CV*s for urinary (Ca; mg), citrate (Cit; mg), chloride (Cl; mmoles), creatinine (Cr; mg), potassium (K; mmoles), sodium (Na; mmoles), magnesium (Mg; mg), oxalate (Ox; mg), phosphorus (P; g), ammonium (NH_4_; mmoles), sulfate (SO_4_; meq), uric acid (UA; g) and urea nitrogen (UN; mg) within a complete population of 9,024 male and 6,758 female kidney stone patients aged 16–80, and in individuals aged 16–45 and 56–80.

	_All_		_Male_		_Female_	
Trait	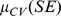	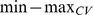	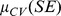	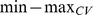	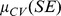	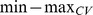
Ca	0.173 (1.11×10^−3^)	0.0–1.91	0.171 (0.00144)	0–1.01	0.176 (0.00175)	3.17×10^−5^ – 1.19
Cit	0.163 (1.35×10^−3^)	2.84×10^−6^ – 1.36	0.160 (0.00171)	2.42×10^−5^ – 1.33	0.166 (0.00218)	2.83×10^−6^ – 1.36
Cl	0.182 (1.20×10^−3^)	2.39×10^−5^ – 1.34	0.178 (0.00156)	3.42×10^−5^ – 1.34	0.188 (0.00187)	2.39×10^−5^ – 1.12
Cr	0.0644 (3.99×10^−4^)	1.19×10^−5^ – 0.212	0.0643 (5.27×10^−4^)	1.19×10^−5^ – 0.212	0.0645 (6.11×10^−4^)	2.53×10^−5^ – 0.212
K	0.129 (8.24×10^−4^)	5.69×10^−6^ – 0.831	0.127 (0.00107)	5.69×10^−6^ – 0.831	0.132 (0.00129)	6.38×10^−6^ – 0.810
Mg	0.152 (9.88×10^−4^)	4.71×10^−6^ – 1.41	0.149 (0.00127)	4.71×10^−6^ – 1.41	0.156 (0.00156)	2.37×10^−5^ – 1.01
Na	0.180 (1.16×10^−3^)	5.97×10^−6^ – 1.17	0.176 (0.00149)	5.98×10^−7^ – 1.13	0.187 (0.00186)	9.14×10^−6^ – 1.17
NH_4_	0.130 (8.87×10^−4^)	4.72×10^−6^ – 1.41	0.128 (0.00112)	4.72×10^−6^ – 1.10	0.133 (0.00143)	2.21×10^−5^ – 1.41
Ox	0.121 (7.87×10^−4^)	4.90×10^−7^ – 0.752	0.119 (0.00102)	4.90×10^−7^ – 0.749	0.122 (0.00123)	4.14×10^−6^ – 0.752
P	0.132 (8.62×10^−4^)	1.81×10^−5^ – 1.41	0.126 (0.00110)	5.67×10^−5^ – 1.41	0.139 (0.00137)	1.81×10^−5^ – 1.29
SO_4_	0.141 (9.34×10^−4^)	3.48×10^−5^ – 1.22	0.136 (0.00118)	3.47×10^−5^ – 1.22	0.147 (0.00151)	9.24×10^−5^ – 1.09
UA	0.108 (7.28×10^−4^)	0.0 – 1.29	0.110 (9.91×10^−4^)	0.0 – 1.29	0.106 (0.00103)	4.77×10^−5^ – 0.871
UN	0.104 (6.61×10^−4^)	0.0 – 1.29	0.103 (8.62×10^−4^)	0.0 – 0.985	0.106 (0.00103)	0.0 – 1.30

CV values approaching zero reflect exact measurements on both sampling days for small numbers of observations.

### Differences in univariate variability by sex

**Figure 1:**
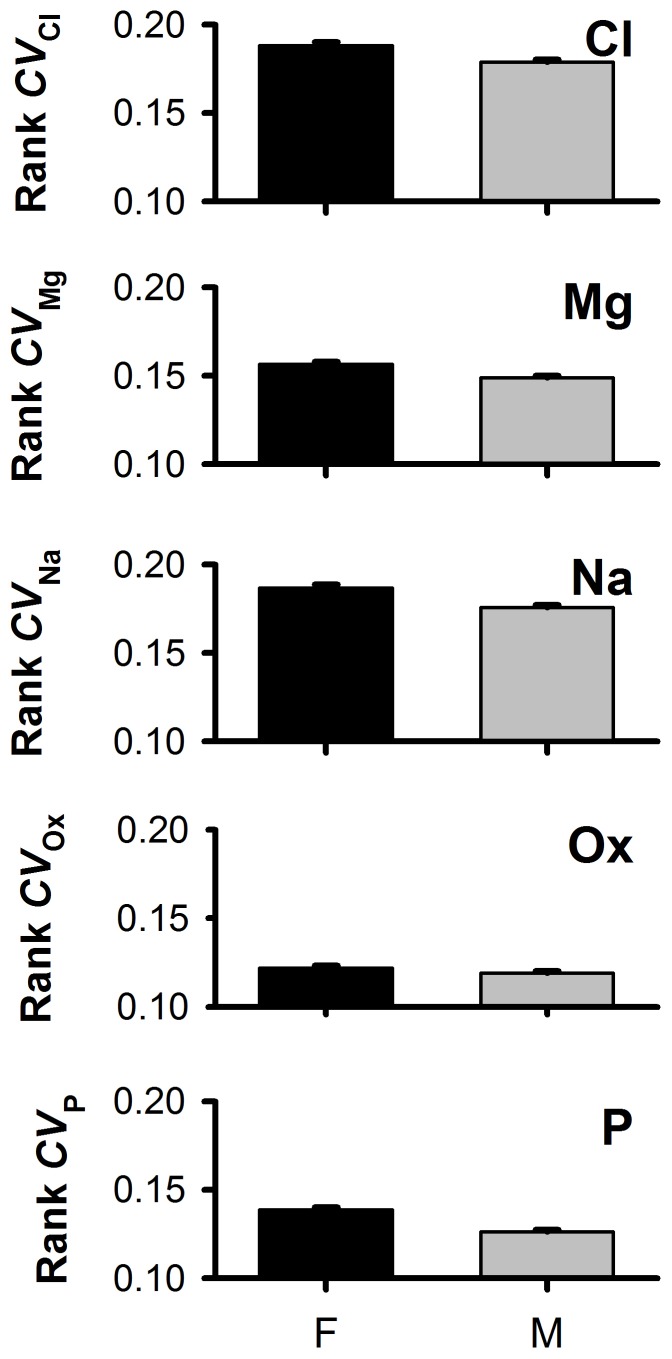
Mean ranked coefficients of variation (*CV*) for chloride (Cl), magnesium (Mg), sodium (Na), oxalate (Ox) and phosphorus (P) in a complete population of 6,758 females and 9,024 males submitted to Litholink, Inc. **significant at nominal (Cl, Mg, Na and Ox) and** ***α*** **_0.05_ False Discovery Rate thresholds selected using backwards stepwise regression (** ***P*** **_acceptance_ = 0.1).** ****

Females had significantly higher 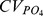 than males across the entire population (Model 1; *P*<0.01) at the FDR (Table 2; Figure 1). Females had higher *CV*s for Mg and Na, and males for Cl and Ox at nominal (*α*_0.05_) *a priori* thresholds, which were not significant after FDR correction (Table 2; Figure 1). Female *CV* was typically from 8–10% higher than in males (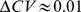). We detected no evidence of sex effects on 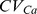 using Model 1 at the FDR threshold or at nominal *a priori* levels of significance (*P*>0.3).

**Table 2:** Effects of gender on ranked coefficients of variation (*CV*) from paired 24-h urinary measurements of urinary ammonium (NH_3_), calcium (Ca), chloride (Cl), citrate (Cit), potassium (K), magnesium (Mg), sodium (Na), oxalate (Ox), phosphorus (P), sulfate (SO_4_), uric acid (UA) and urine urea nitrogen (UN) adjusted for creatinine, urinary volume, age and weight in the complete population of 6,758 females and 9,024 males aged 16–80, and in individuals aged 16–45 and 56–80 in a stepwise backward regression (*P*_acceptance_≤0.1).

	All				16–45				56–80			
Trait (units)	*β*_sex_ (SE)	*F*	*P_nom_*	*r* ^2^	*β*_sex_ (SE)	*F*	*P*_nom_	*r* ^2^	*β*_sex_ (SE)	*F*	*P*_nom_	*r* ^2^
Ca	−72.8 (81.9)	0.79	0.3745	0.0673	−126.8 (126.0)	1.01	0.3145	0.0496	144.2 (133.0)	1.18	0.2782	0.0907
Cit	12.0 (83.6)	0.02	0.8861	0.0263	399.0 (124.7)	10.2	0.0014*	0.0268	−476.0 (133.5)	12.7	0.0002**	0.0367
Cl	−162.5 (72.1)	5.08	0.0242	0.114	−99.7 (111.3)	0.80	0.3702	0.0888	−297.8 (125.8)	5.60	0.0180	0.1364
Cr	−116.3 (81.0)	2.06	0.1508	0.0149	−36.3 (128.2)	0.08	0.7769	0.0124	−230.7 (134.4)	2.95	0.0861	0.0096
K	132.4 (80.2)	2.72	0.0990	0.0487	285.0 (120.0)	5.67	0.0173	0.0390	−52.7 (131.7)	0.16	0.6893	0.0534
Mg	−186.3 (81.0)	5.29	0.0215	0.0285	−139.0 (129.0)	1.16	0.2816	0.0191	−365.4 (137.8)	7.03	0.0080^†^	0.0364
Na	−155.0 (75.7)	4.19	0.0406	0.116	−168.7 (111.6)	2.28	0.1307	0.0913	−178.9 (125.0)	2.05	0.1525	0.1363
NH_4_	42.0 (83.7)	0.25	0.6165	0.0230	−62.6 (130.6)	0.23	0.6318	0.0194	132.8 (132.9)	1.00	0.3178	0.0299
Ox	184.0 (80.8)	5.19	0.0227	0.0332	16.3 (130.6)	0.02	0.9009	0.0278	287.1 (140.0)	4.21	0.0403	0.0442
P	−278.6 (74.1)	14.1	0.0002**	0.0694	−207.6 (116.6)	3.17	0.0752	0.0545	−295.9 (129.2)	5.25	0.0220	0.0750
SO_4_	−123.9 (79.1)	2.45	0.1174	0.0348	29.9 (125.2)	0.06	0.8114	0.0314	−166.6 (137.3)	1.47	0.2253	0.0363
UA	85.7 (78.2)	1.20	0.2728	0.0937	6.20 (125.5)	0.0	0.9606	0.0863	45.4 (139.7)	0.11	0.7455	0.1065
UN	−60.0 (72.8)	0.68	0.4098	0.0971	−6.28 (123.8)	0.0	0.9596	0.0865	−98.7 (125.6)	0.62	0.4321	0.1210

Significance (below) is corrected for multiple observations via False Discovery Rate [Verhoeven, 2005 #22836]. Nominal *P*-value (*P*_nom_) is reported at acceptance or removal from the model during the stepwise procedure. Units indicates original values used in *CV* calculation.

*P*_nom_ = nominal *P*-value.

* *P*_FDR_<0.05,

** *P*_FDR_<0.01.

Correlation coefficients (*r* ^2^) refer to the complete model correlation, or model fit at rejection of nonsignificant terms via backwards regression (removal at *P*>0.1).

Negative *β* indicates lower female *CV*; positive *β* higher male CV.

### Changes in *CV*s by major life-history category

**Figure 2:**
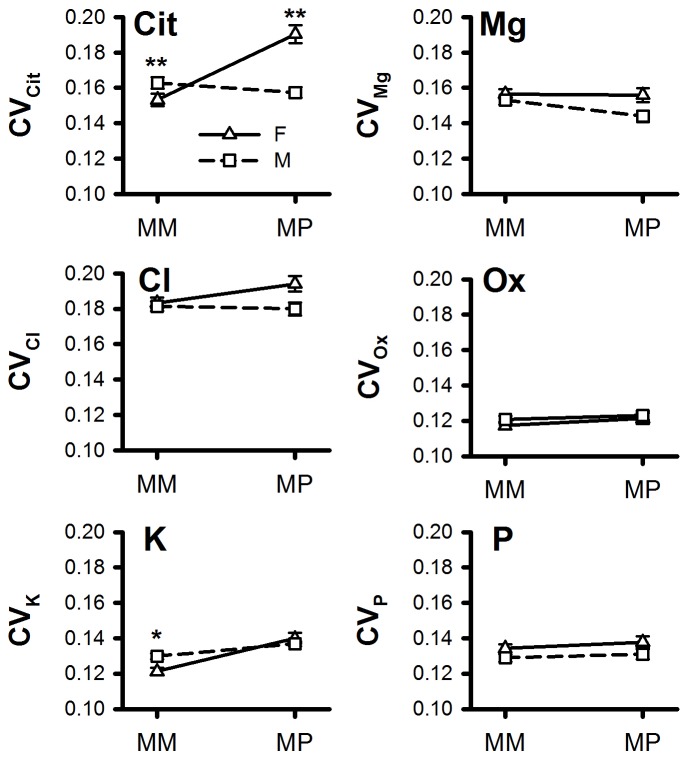
Mean ranked coefficients of variation (*CV*) for citrate (Cit), chloride (Cl), potassium (K), magnesium (Mg) and phosphorus (P) in 4097 females (F) and 4014 males (M) in an approximate ‘menarche-menstrual’ (MM) period (ages 16–45) and in 1715 females and 3479 males in an approximate ‘postmenopausal’ group (PM) (ages 56–80). Significance of differences in *CV* by trait and age group (MM *vs*. PM) indicated as *P*<0.05*, *P*<0.01**.

A total of 4,128 males and 4,219 females from ages 16–45 and 3,578 male and 1,772 females were available. We detected marked changes in the pattern of male and female *CV*s in in the subdivided Model 1 for the examination of sex effects on variation at ages 16–45 and 56–80. Males had significantly higher 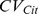 from the ages of 16–45 years compared to females (*P*<0.01) and females significantly higher 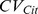 than males from 56–80 years at the FDR (*P*<0.01) (Table 2; Figure 2). From 16–45 years, males had significantly higher 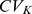 (*P*<0.01) at the nominal significance threshold but not after multiple correction.

Reminiscent of Model 1, females had higher 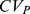 from 56–80 years and marginally higher 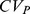 from 16–45 at the nominal significance threshold. Females also had nominally higher 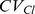 and 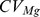 than males in the 56–80 year age group while 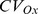 was nominally higher in males. None of these associations was significant after multiple correction (*P*_FDR_>0.05); 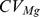 was marginally higher in females at the FDR in the 56–80 age category (*P*<0.1) (Table 2; Figure 2).

Using partial regression covariates identified in Model 1, we detected significant effects of sex×age class interaction on 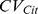 at the FDR threshold (*P*<0.001) between the two age groups (16–45 years and 56–80 years). There were no significant sex×age group effect for any other urinary metabolite after multiple correction (*P*_FDR_>0.1) (Table 3).

**Table 3:** Effects of sex-by-age category (16–45 years *vs*. 56–80 years) interaction on ranked coefficients of variation (*CV* = *σ/μ*) calculated from original paired 24-h urinary solutes (urinary ammonium (NH_3_), calcium (Ca), chloride (Cl), citrate (Cit), potassium (K), magnesium (Mg), sodium (Na), oxalate (Ox), phosphorus (P), sulfate (SO_4_), uric acid (UA), urine urea nitrogen (UN)) collected from 6,758 female and 9,024 male stone-formers aged 16–80 at the False Discovery Rate [Verhoeven, 2005 #22836].

Trait	*F*	*P_nom_*	*r* ^2^
Ca	1.70	0.1920	0.0682
Cit	29.2	<0.0001**	0.0293
Cl	1.97	0.1601	0.108
Cr	1.98	0.1590	7.65×10^−4^
K	5.34	0.0208	0.0521
Mg	3.83	0.0503	0.0307
Na	0.18	0.6728	0.111
NH_4_	0.43	0.5116	0.0225
Ox	0.02	0.8932	0.0342
P	0.28	0.5924	0.0678
SO_4_	0.55	0.4574	0.0354
UA	0.04	0.8466	0.0941
UN	0.11	0.7389	0.0988

## Discussion

Females had higher coefficients of variation for urinary phosphorus over the entire age distribution and for citrate from 56–80 years, while males had higher *CV* for citrate from 16–45 years of age. We did not detect male-female differences for *CV*s in urinary calcium despite predictions from our rodent model [13]. Other studies have found diurnal [25], inter- and/or intra-individual variation in urine solutes including calcium and oxalate [2], [3], [4], [5], [7], [8], [26] exhibit high variability, including in stone-formers [6], but sex effects on residual variation in urinary solutes are a novel finding.

Since our study did not employ dietary controls or journaling, our findings might result from day-to-day male-female dietary variability, male∶female genetic or endocrine differences, or differences in the fidelity of urine collections; sex-by-strain and sex-by-genotype effects in our rodent model were detected on controlled, constant diets [13]. Sulfate is encountered in many food additives [27] and while we included urinary nitrogen as a dietary control variable for protein intake (see [22]) there are numerous dietary sources of phosphorus such as dark sodas and preserved fast foods [28] besides meat and dairy products [29] which might create day-to-day variability in urinary phosphorus. Our results may reflect changing output from transient dietary preference, largely in females, or integral differences in residual variance between the sexes in urinary solutes. We cannot be certain, therefore, that human residual variance in urinary solutes represents the same phenomenon we document in our rodent model. No ethnic variables were available; our findings thus may represent a ‘generalized’ trend within stone-formers.

Whether resulting from dietary variability or endogenous endocrine or genetic effects, IIV in urinary solutes may have significant diagnostic consequences: Parks et al [9] found that single measurements were insufficient for the analysis of urinary risk factors for kidney stones, with daily assays varying by a standard deviation (*σ*) or more. Our rodent work suggests that IIV can bias estimation of ‘classical’ genetic effects on means: a region of chromosome 1 associated with *CV* in urinary calcium in our rodent model (‘*hypercalciuria coefficient of variation 1*’) obscured the detection of a nearby locus with effects on mean urinary calcium (‘*hypercalcuria 1*’) [30]. We have also detected sex-by-genotype and sex-by-strain effects on residual variance in urinary calcium using genetic mapping in rodents, suggesting sex linkage of these effects [13] and other results of ours indicate a heritability of 7% for 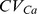 in humans and 60% in rats (Perry et al unpubs). Sex might similarly affect the measurement of effects on renal solutes, whether as a heritable effect or a general male-female difference in dietary consistency. Differences in reporting accuracy might be excluded as a possible cause for our findings, since sex effects on *CV* were only detected for two urinary solutes. Notably, females did have significantly higher *CV*s for urinary phosphorus. Phosphorus and calcium are physiologically related [31]; dietary phosphorus affects urinary calcium and calcium balance [32], [33]. Phosphorus was not assayed in our rodent work, but it is possible that sex-based differences in urinary calcium might be dependent on countercurrent IIV in urinary phosphorus.

Male *CV*s for urinary citrate were higher than in females in the menarche-menopause group, and females higher at post-menopausal ages. Both female humans and rats excrete more citrate than males overall [34], [35], [36], which may confer some protection against nephrolithiasis in females [37]. Assuming that our sample was representative within our defined age categories, our results might indicate periodic endogenous or dietary changes in relative citrate protection between the sexes depending on age. Female stone incidence is higher than in males at greater ages in many populations [38].

### Conclusions

Exogenous and endogenous sex hormones have major main effects on renal physiology [39]; estrogen decreases urinary oxalate, urinary calcium and crystal deposition while testosterone promotes stone formation by suppressing renal osteopontin and increasing urinary oxalate [40]. Men experience more stones and produce more urinary phosphate, calcium, uric acid, magnesium, oxalate and creatinine and less citrate than women of the same age [39], [41], [42]. Our findings indicate that males and females also have differences in IIV for citrate and phosphorus with changing IIV for citrate by age and sex age, either as a function of dietary differences or from endogenous effects associated with sex. These differences might affect the reliability of individual phenotype in genetic mapping assays, alter estimates of basal physiology, or even cause transient declines in protective metabolites.

## Supporting Information

Table S1 **Final models for partial regression terms in stepwise backward regression (** ***P*** **_acceptance_≤0.1) of ranked coefficients of variation (** ***CV*** **) for 24-h urinary ammonium (NH_3_), calcium (Ca), chloride (Cl), citrate (Cit), potassium (K), magnesium (Mg), sodium (Na), oxalate (Ox), phosphorus (P), sulfate (SO_4_), uric acid (UA) and urine urea nitrogen (UN) in 9,024 male and 6,758 female stone-formers aged 16–80, and in age categories 16–45 years and 56–80 years.** Partial regression terms were removed at nominal *P*-value (*P*_nom_)>0.1.
(DOC)
