# Supplementary material for: Effects of Sex on Intra-Individual Variance in Urinary Solutes in Stone-Formers Collected from a Single Clinical Laboratory
Source: PLoS One. 2013 Jun 19;8(6):e53637. doi: 10.1371/journal.pone.0053637 (PMC3686766; doi:10.1371/journal.pone.0053637)
Supplement: Table S1 — Final models for partial regression terms in stepwise backward regression ( P acceptance≤0.1) of ranked coefficients of variation ( CV ) for 24-h urinary ammonium (NH3), calcium (Ca), chloride (Cl), citrate (Cit), potassium (K), magnesium (Mg), sodium (Na), oxalate (Ox), phosphorus (P), sulfate (SO4), uric acid (UA) and urine urea nitrogen (UN) in 9,024 male and 6,758 female stone-formers aged 16–80, and in age categories 16–45 years and 56–80 years. Partial regression terms were removed at nominal P-value (P nom)>0.1. (DOC) [file pone.0053637.s001.doc]

**Table S1. Final models for partial regression terms in stepwise backward regression (*P*_acceptance_ ≤ 0.1) of ranked coefficients of variation (*CV*) for 24-h urinary ammonium (NH_3_), calcium (Ca), chloride (Cl), citrate (Cit), potassium (K), magnesium (Mg), sodium (Na), oxalate (Ox), phosphorus (P), sulfate (SO_4_), uric acid (UA) and urine urea nitrogen (UN) in 9,024 male and 6,758 female stone-formers aged 16-80, and in age categories 16-45 years and 56-80 years. Partial regression terms were removed at nominal *P*-value (*P*_nom_) > 0.1.**

| Group | Urinary solute | Abbr | Model |
| --- | --- | --- | --- |
| All | Calcium | Ca | $\beta_{age}X_{age}+\beta_{CV_{Cr}}X_{CV_{Cr}}+\beta_{CV_{vol}}X_{CV_{vol}}+\beta_{CV_{Na}}X_{CV_{Na}}+\beta_{CV_{UUN}}X_{CV_{UUN}}$ |
|  | Citrate | Cit | $\beta_{wt}^{2}X_{wt}^{2}+\beta_{CV_{Cr}}X_{CV_{Cr}}+\beta_{CV_{vol}}X_{CV_{vol}}$ |
|  | Chloride | Cl | $\alpha_{sex}+\beta_{age}^{2}X_{age}^{2}+\beta_{CV_{Cr}}X_{CV_{Cr}}+\beta_{CV_{vol}}X_{CV_{vol}}$ |
|  | Creatinine | Cr | $\beta_{wt}X_{wt}$ |
|  | Potassium | K | $\alpha_{sex}+\beta_{wt}X_{wt}+\beta_{age}X_{age}+\beta_{CV_{Cr}}X_{CV_{Cr}}+\beta_{CV_{vol}}X_{CV_{vol}}$ |
|  | Magnesium | Mg | $\alpha_{sex}+\beta_{wt}X_{wt}+\beta_{age}^{2}X_{age}^{2}+\beta_{CV_{Cr}}X_{CV_{Cr}}+\beta_{CV_{vol}}X_{CV_{vol}}$ |
|  | Sodium | Na | $\alpha_{sex}+\beta_{age}^{2}X_{age}^{2}+\beta_{CV_{Cr}}X_{CV_{Cr}}+\beta_{CV_{vol}}X_{CV_{vol}}$ |
|  | Ammonium | NH_4_ | $\beta_{age}X_{age}+\beta_{CV_{Cr}}X_{CV_{Cr}}+\beta_{CV_{vol}}X_{CV_{vol}}$ |
|  | Oxalate | Ox | $\alpha_{sex}+\beta_{wr}X_{wt}+\beta_{age}^{2}X_{age}^{2}+\beta_{CV_{Cr}}X_{CV_{Cr}}+\beta_{CV_{vol}}X_{CV_{vol}}$ |
|  | Phosphorus | P | $\alpha_{sex}+\beta_{age}X_{age}+\beta_{CV_{Cr}}X_{CV_{Cr}}+\beta_{CV_{UUN}}X_{CV_{UUN}}$ |
|  | Sulfate | SO_4_ | $\beta_{wt}^{2}X_{wt}^{2}+\beta_{age}^{2}X_{age}^{2}+\beta_{CV_{Cr}}X_{CV_{Cr}}+\beta_{CV_{vol}}X_{CV_{vol}}$ |
|  | Uric acid | UA | $\beta_{wt}X_{wt}+\beta_{age}X_{age}+\beta_{CV_{Cr}}X_{CV_{Cr}}+\beta_{CV_{vol}}X_{CV_{vol}}$ |
|  | Urine urea nitrogen | UN | $\beta_{age}^{2}X_{age}^{2}+\beta_{CV_{Cr}}X_{CV_{Cr}}+\beta_{CV_{vol}}X_{CV_{vol}}$ |
| 16-45 years | Calcium | Ca | $\beta_{wt}X_{wt}+\beta_{age}^{2}X_{age}^{2}+\beta_{CV_{vol}}X_{CV_{vol}}+\beta_{CV_{Na}}X_{CV_{Na}}+\beta_{CV_{UUN}}X_{CV_{UUN}}$ |
|  | Citrate | Cit | $\alpha_{sex}+\beta_{wt}X_{wt}+\beta_{age}X_{age}+\beta_{CV_{Cr}}X_{CV_{Cr}}+\beta_{CV_{vol}}X_{CV_{vol}}$ |
|  | Chloride | Cl | $\beta_{CV_{Cr}}X_{CV_{Cr}}+\beta_{CV_{vol}}X_{CV_{vol}}$ |
|  | Creatinine | Cr | $\beta_{wt}X_{wt}+\beta_{CV_{vol}}X_{CV_{vol}}$ |
|  | Potassium | K | $\alpha_{sex}+\beta_{age}X_{age}+\beta_{CV_{Cr}}X_{CV_{Cr}}+\beta_{CV_{vol}}X_{CV_{vol}}$ |
|  | Magnesium | Mg | $\beta_{wt}X_{wt}+\beta_{age}X_{age}+\beta_{age}^{2}X_{age}^{2}+\beta_{CV_{Cr}}X_{CV_{Cr}}+\beta_{CV_{vol}}X_{CV_{vol}}$ |
|  | Sodium | Na | $\beta_{CV_{Cr}}X_{CV_{Cr}}+\beta_{CV_{vol}}X_{CV_{vol}}$ |
|  | Ammonium | NH_4_ | $\beta_{age}X_{age}+\beta_{CV_{Cr}}X_{CV_{Cr}}+\beta_{CV_{vol}}X_{CV_{vol}}$ |
|  | Oxalate | Ox | $\beta_{age}X_{age}+\beta_{age}^{2}X_{age}^{2}+\beta_{CV_{Cr}}X_{CV_{Cr}}+\beta_{CV_{vol}}X_{CV_{vol}}$ |
|  | Phosphorus | P | $\alpha_{sex}+\beta_{age}X_{age}+\beta_{CV_{Cr}}X_{CV_{Cr}}+\beta_{CV_{UUN}}X_{CV_{UUN}}$ |
|  | Sulfate | SO_4_ | $\beta_{wt}^{2}X_{wt}^{2}+\beta_{CV_{Cr}}X_{CV_{Cr}}+\beta_{CV_{vol}}X_{CV_{vol}}$ |
|  | Uric acid | UA | $\beta_{wt}X_{wt}+\beta_{CV_{Cr}}X_{CV_{Cr}}+\beta_{CV_{vol}}X_{CV_{vol}}$ |
|  | Urine urea nitrogen | UN | $\beta_{CV_{Cr}}X_{CV_{Cr}}+\beta_{CV_{vol}}X_{CV_{vol}}$ |
| 56-80 years | Calcium | Ca | $\beta_{wt}^{2}X_{wt}^{2}+\beta_{age}X_{age}+\beta_{CV_{Cr}}X_{CV_{Cr}}+\beta_{CV_{vol}}X_{CV_{vol}}+\beta_{CV_{Na}}X_{CV_{Na}}+\beta_{CV_{UUN}}X_{CV_{UUN}}$ |
|  | Citrate | Cit | $\alpha_{sex}+\beta_{age}X_{age}+\beta_{CV_{Cr}}X_{CV_{Cr}}+\beta_{CV_{vol}}X_{CV_{vol}}$ |
|  | Chloride | Cl | $\alpha_{sex}+\beta_{age}^{2}X_{age}^{2}+\beta_{CV_{Cr}}X_{CV_{Cr}}+\beta_{CV_{vol}}X_{CV_{vol}}$ |
|  | Creatinine | Cr | $\alpha_{sex}+\beta_{CV_{vol}}X_{CV_{vol}}$ |
|  | Potassium | K | $\beta_{wt}^{2}X_{wt}^{2}+\beta_{age}X_{age}+\beta_{CV_{Cr}}X_{CV_{Cr}}+\beta_{CV_{vol}}X_{CV_{vol}}$ |
|  | Magnesium | Mg | $\alpha{}_{sex}+\beta_{wt}X_{wt}+\beta_{age}X_{age}+\beta_{CV_{Cr}}X_{CV_{Cr}}+\beta_{CV_{vol}}X_{CV_{vol}}$ |
|  | Sodium | Na | $\beta_{CV_{Cr}}X_{CV_{Cr}}+\beta_{CV_{vol}}X_{CV_{vol}}$ |
|  | Ammonium | NH_4_ | $\beta_{CV_{Cr}}X_{CV_{Cr}}+\beta_{CV_{vol}}X_{CV_{vol}}$ |
|  | Oxalate | Ox | $\alpha_{sex}+\beta_{wt}X_{wt}+\beta_{age}X_{age}+\beta_{CV_{Cr}}X_{CV_{Cr}}+\beta_{CV_{vol}}X_{CV_{vol}}$ |
|  | Phosphorus | P | $\alpha_{sex}+\beta_{age}^{2}X_{age}^{2}+\beta_{CV_{Cr}}X_{CV_{Cr}}+\beta_{CV_{vol}}X_{CV_{vol}}+\beta_{CV_{UUN}}X_{CV_{UUN}}$ |
|  | Sulfate | SO_4_ | $\beta_{wt}^{2}X_{wt}^{2}+\beta_{CV_{Cr}}X_{CV_{Cr}}+\beta_{CV_{vol}}X_{CV_{vol}}$ |
|  | Uric acid | UA | $\beta_{wt}X_{wt}+\beta_{CV_{Cr}}X_{CV_{Cr}}+\beta_{CV_{vol}}X_{CV_{vol}}$ |
|  | Urine urea nitrogen | UN | $\beta_{age}^{2}X_{age}^{2}+\beta_{CV_{Cr}}X_{CV_{Cr}}+\beta_{CV_{vol}}X_{CV_{vol}}$ |
